# An international multi-site, randomized controlled trial of a mindfulness-based psychoeducation group programme for people with schizophrenia

**DOI:** 10.1017/S0033291717000526

**Published:** 2017-04-04

**Authors:** W. T. Chien, D. Bressington, A. Yip, T. Karatzias

**Affiliations:** 1 Mental Health Care Research Group, School of Nursing, Faculty of Health and Social Sciences, The Hong Kong Polytechnic University, Hung Hom, Hong Kong, People's Republic of China; 2 School of Health & Social Care, Edinburgh Napier University, Edinburgh, UK

**Keywords:** Mindfulness-based interventions, psychoeducation, randomized controlled trials, re-hospitalization, schizophrenia spectrum disorders

## Abstract

**Background:**

We aimed to test a mindfulness-based psychoeducation group (MBPEG), *v.* a conventional psychoeducation group (CPEG) *v.* treatment as usual (TAU), in patients with schizophrenia-spectrum disorders over a 24-month follow-up.

**Method:**

This single-blind, multi-site, pragmatic randomized controlled trial was conducted in six community treatment facilities across three countries (Hong Kong, mainland China and Taiwan). Patients were randomly allocated to one of the treatment conditions, and underwent 6 months of treatment. The primary outcomes were changes in duration of re-hospitalizations and mental state (Positive and Negative Syndrome Scale; PANSS) between baseline and 1 week, and 6, 12 and 18 months post-treatment.

**Results:**

A total of 300 patients in each country were assessed for eligibility between October 2013 and 30 April 2014, 38 patients per country (*n* = 342) were assigned to each treatment group and included in the intention-to-treat analysis. There was a significant difference in the length of re-hospitalizations between the three groups over 24 months (*F*_2,330_ = 5.23, *p* = 0.005), with MBPEG participants having a shorter mean duration of re-hospitalizations than those in the other groups. The MBPEG and CPEG participants had significant differential changes in proportional odds ratios of complete remission (all individual PANSS items <3) over the 24-month follow-up (37 and 26%, respectively), as opposed to only 7.2% of the TAU group (χ^2^ = 8.9 and 8.0, *p* = 0.001 and 0.003, relative risk = 3.5 and 3.1, 95% confidence interval 2.0–7.2 and 1.6–6.3).

**Conclusions:**

Compared with TAU and CPEG, MBPEG improves remission and hospitalization rates of people with schizophrenia spectrum disorders over 24 months.

## Introduction

Schizophrenia is a severe mental disorder with a global 12-month prevalence of 1.1–1.8% of adult populations (Global Burden of Disease Study 2013 Collaborators, 2015). Antipsychotic medications are evidenced to reduce positive symptoms but cannot significantly improve functioning, risk of relapse and residual symptoms of most psychotic patients (Bhagyavathi *et al.* 2015; Schennach *et al*. 2015). According to recent practice guidelines (Lehman *et al.* 2004; National Institute for Health and Care Excellence, 2014), psychosocial interventions such as psychoeducation and other similar programmes can improve patient outcomes when used in adjunct to antipsychotic pharmacotherapy. A combination of medications and psychosocial interventions for people with early psychosis has been shown to improve these patients’ functioning and quality of life, as well as to reduce their risk of relapse, when compared with pharmacological treatment alone (Guo *et al.* 2010).

Systematic reviews of psychoeducation interventions for schizophrenia show that the intervention when delivered in both one-to-one and group sessions significantly reduces relapse rates and improves levels of medication adherence (Xia *et al.* 2011). However, many psychoeducation intervention programmes for people with schizophrenia do not incorporate adequate strategies for empowering self-management of the illness and pay scant attention towards facilitating an acceptance of the illness and the distressing experiences of symptoms (Bäuml *et al.* 2006; Chadwick *et al.* 2009).

Mindfulness-based stress reduction (MBSR) programmes consist of approaches that aim to facilitate patients’ acceptance, and are focused on reducing distress by changing negative thoughts, emotions and attitudes towards the illness (Chadwick *et al.* 2009; Chien & Lee, 2013). Research evidence regarding the effectiveness of MBSR on stress and illness management in patients with depression and anxiety is prolific and well established (Ma & Teasdale, 2004). Standard biweekly 10-session MBSR programmes for severe depression and anxiety are evidenced to empower patients’ self-care and symptom management, improve insight and enhance control over distressing thoughts (Ma & Teasdale, 2004; Chlesa & Serretti, 2011). The benefits of mindfulness-based interventions (MBI) have also been evidenced in the treatments of a wide variety of physical and mental disorders (Pull, 2009; Chlesa & Serretti, 2011). It has been suggested that the positive effects of MBI in major depression could be attributed to reductions of over-general memory and ruminative thinking, and improvements in meta-awareness leading to an overall decrease in depression severity (Williams *et al.* 2000). The findings of other similar studies in depressive/anxiety disorders also suggested that MBI training could enhance patients’ ability to reflect upon previous crises in a decentred manner, which helps to relate differently to earlier distressing experiences in order to prevent future relapse (Kingston *et al.* 2007; Hargus *et al.* 2010).

The approach could be particularly useful in schizophrenia spectrum disorders as they are often partially responsive to current treatments; resulting in frequent relapses with associated high societal and economic burden (Chlesa & Serretti, 2011). However, research into the effectiveness of MBI as treatments for psychotic disorders is still in its relatively early stages. Two recent feasibility trials (Freeman *et al.* 2002; Chadwick *et al.* 2009) with short follow-up suggested that there might be benefits from MBI (eight to 10 biweekly sessions) for psychosis in terms of improving illness management and preventing relapses, and another recent single-site controlled trial in Hong Kong (Chien & Lee, 2013) found that a mindfulness-based psychoeducation programme for 36 patients with schizophrenia resulted in significant improvements in insight into the illness, functioning and symptom severity over a longer-term follow-up. An earlier meta-analysis (Khoury *et al.* 2013), including 13 studies with 468 patients with psychotic disorders, found an overall moderate effect of MBI on symptom severity and relapse rate in single-group pre- and post-test studies, and a small to moderate effect in studies using a comparison group. Our recent systematic review of MBI for schizophrenia (Lam & Chien, 2016) identified six eligible randomized controlled trials (RCTs) which described benefits in schizophrenia (i.e. improvements in global functioning, emotional regulation and relapse prevention). Because of variation in study designs, outcome measures and interventions, further RCTs are needed in order to be confident of the positive effects of MBI for diverse samples with schizophrenia spectrum disorders. Therefore, these preliminary findings suggest that a multi-site RCT with a larger sample and active comparison group and longer follow-ups is required in order to robustly test the efficacy of MBI for schizophrenia.

To the best of our knowledge, this is the first international multi-site study testing a mindfulness-based psychoeducation group (MBPEG) programme for out-patients with schizophrenia spectrum disorders. We hypothesized, on the basis of accumulating evidence (Ma & Teasdale, 2004; Kingston *et al.* 2007; Chadwick *et al.* 2009; Chlesa & Serretti, 2011; Chien & Lee, 2013), that the effect of MBPEG in addition to treatment as usual (TAU) would result in significantly greater improvement of patients’ primary outcomes (re-hospitalizations and symptoms), as well as insight into illness/treatment and functioning over a 24-month follow-up, when compared with those in a conventional psychoeducation group (CPEG) programme or TAU alone.

## Method

### Trial registration

This trial was prospectively registered at ClinicalTrials.gov (NCT01667601) (https://clinicaltrials.gov/ct2/show/NCT01667601).

### Study design

The study was a single-blind multi-centre RCT with a three-group repeated-measures design comparing the treatment outcomes of out-patients with schizophrenia spectrum disorders. The study was approved by the Human Subjects Research Ethics Committee at The Hong Kong Polytechnic University (HSEARS201211001) and the clinics under study.

### Participants

Patients were recruited from and assessed at psychiatric out-patient clinics in Hong Kong, Taiwan and China (i.e. two clinics in each country). At recruitment, about 1000 patients with schizophrenia spectrum disorders were attending the clinics in each country. Inclusion criteria for participants from the six clinics were: age 18–64 years; current Diagnostic and Statistical Manual of Mental Disorders (DSM-IV) diagnosis of schizophrenia and other psychotic disorders as ascertained by the Structured Clinical Interview for DSM-IV Axis I Disorders (SCID-I) (First *et al.* 2001); having ⩽5years of illness at recruitment; and able to understand Chinese/Mandarin. Exclusion criteria were: having co-morbidity of another mental illness such as affective and organic brain disorders; moderate or severe learning disability; and receiving mindfulness and/or other psychotherapies in the past 2 years. All patients provided their voluntary written informed consent to participate after receiving a full description of the study.

### Randomization and masking

**Fig. 1.**
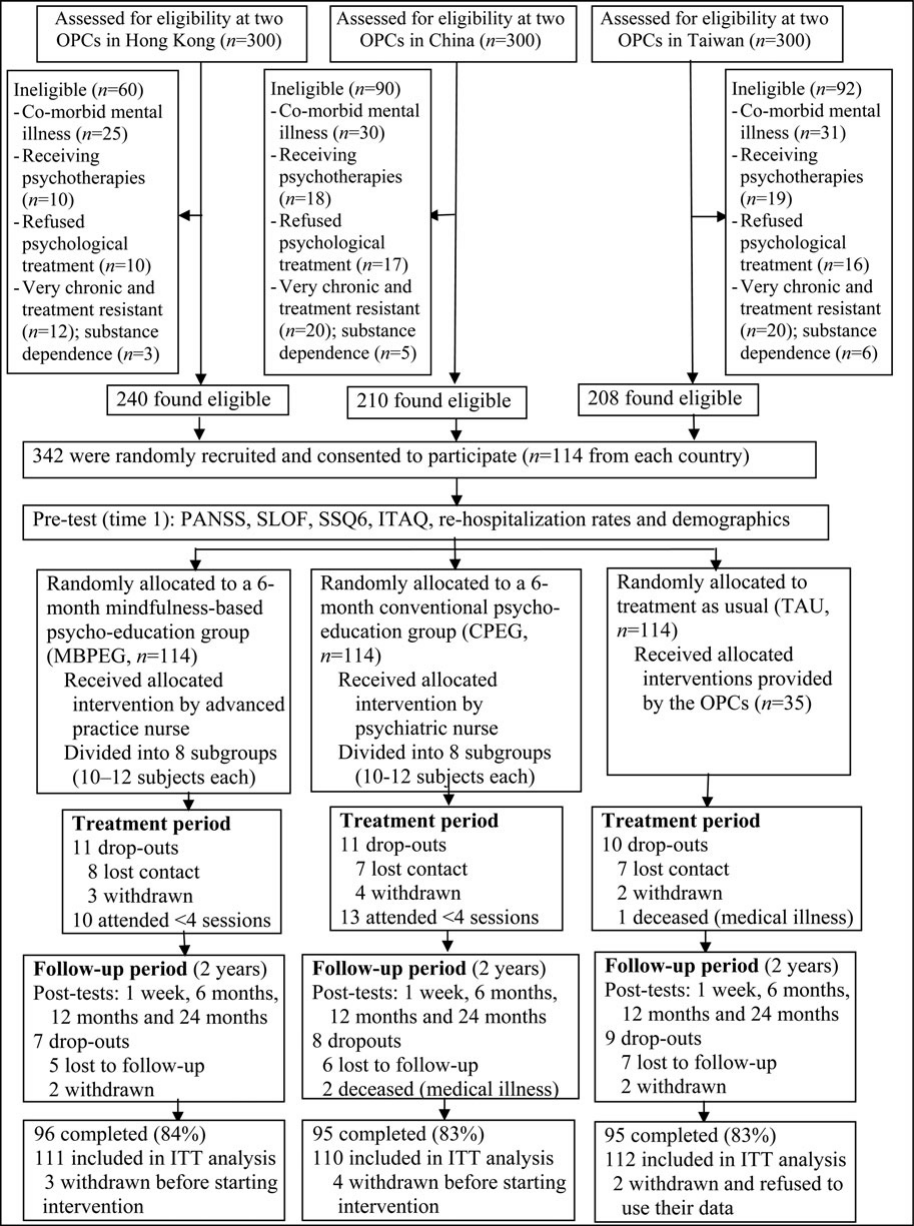
Consolidated Standards of Reporting Trials (CONSORT) diagram of clinical trial for two treatment and usual-care groups. OPC, Out-patient clinic; PANSS, Positive and Negative Syndrome Scale; SLOF, Specific Level of Functioning Scale; SSQ6, six-item Social Support Questionnaire; ITAQ, Insight and Treatment Attitudes Questionnaire; ITT, intention-to-treat.

The consenting patients were listed in alphabetical order of their surnames, each was assigned a unique study number and 114 (48–55%) from each country were then selected randomly from the lists, using computer-generated random numbers (using www.random.org) by an independent statistician. Following baseline measurement at the clinics, randomization to a 6-month course of MBPEG plus TAU, CPEG plus TAU, or TAU alone (38 patients per treatment group per country) was conducted by an independent statistician off-site using a stochastic minimization program to balance the gender, severity of psychotic symptoms [scores of all items of Positive and Negative Syndrome Scale (PANSS) ⩽3, total score of 60–150, or total score of +120] (Bell *et al.* 1992), and medication adherence (poor or satisfactory on the Adherence Rating Scale) (Coldman *et al.* 2002) (see Fig. 1 for the flow of participants in the study). To minimize potential contamination of treatment effects, or subject biases, participants were asked not to reveal their study participation to clinical staff or discuss the contents of their programme with other patients in the clinics who were not in their treatment group. The participant lists were also concealed from the clinic staff and researchers over the duration of the study. The outcome assessor and clinic staff were blinded to treatment allocation.

### Procedures

Participants (*n* = 114) received a 24-week (12 biweekly, 2-h sessions) MBPEG, consisting of 10–12 patients per group. The MBPEG was based on Kabat-Zinn's MBSR programme modified for Chinese psychotic patients in Hong Kong (Chien & Thompson, 2014), as well as the recent psychoeducation programme by Chan *et al.* (2009). One of the six trained psychiatric advanced practice nurses (average clinical and mindfulness training experiences: 8 to 10 and 2 to 3 years, respectively) worked with patients to help them become increasingly aware of and relate differently to their thoughts, feelings, perceptions and physical sensations, as opposed to understanding them as being accurate readouts on reality. The mindfulness theory employed in MBPEG assumes that, in patients with psychotic disorders, a lack of insight into the illness and its treatment and inability of accepting and/or resolving the illness-related problems, together with poor psychosocial functioning, impair help-seeking and illness management behaviours. The MBPEG is designed to gradually increase patients’ insight into and acceptance of their illness, and be motivated to control their psychotic symptoms and actively engage with their treatment plans. The programme consisted of seven domains in three phases (see Table 1). Individual participants were requested and encouraged to perform regular (at least twice daily) practices of focused awareness of thoughts, feelings, bodily sensations and mindful breathing and walking in the early stages; and then, they were facilitated to establish self-empowering and constructive outlooks for working with distressing, or negative thoughts and feelings.

**Table 1.** Mindfulness-based psychoeducation programme for people with schizophrenia

Phase	Component	Goals or rationale	Main topics/themes	Practice
I.	1. Orientation and engagement (two sessions)	a. Establishment of mutual trust and respect, treatment goals and objectives, and expected roles and responsibilities in the group; and b. Understanding of the group programme and information about the illness and its symptoms	• Orientation to the MBPEG and its functions • Establishing trust and respect among group members • Achieving agreed goals and objectives • Schizophrenia and its impacts	i. Self-introduction and game activities ii. Group discussion about their roles and responsibilities in the group, and about schizophrenia and its impacts on patients and their families
	2. Focused awareness of bodily sensations, thoughts, feelings and symptoms (three sessions)	Session 1: Stepping out of automatic pilot and negative thoughts Rationale: a. Mindfulness starts when we recognize the tendency to be on automatic pilot; b. Commitment to learning how to step out of it and being aware of each symptom and related experience; and c. Practice in purposefully drawing attention to bodily sensations and movements	• Body scan, noticing sensations, feelings and thoughts • Dealing with barriers to focusing thoughts, emotions and events, particularly pleasurable events	i. Body scan ii. Breath and awareness and mindfulness thereof iii. Focusing on both pleasant and annoying events iv. Focused awareness of the body, thoughts and feelings (homework)
		Session 2: Mindfulness of the breath and staying present Rationale: a. Becoming familiar with the behaviour of the mind (often being busy and scattered); b. The mind is most scattered when trying to cling to something and avoid others; and c. Mindfulness offers a means to stay present by providing another place from which to view things	• Awareness of the breath offers an anchor to the present (a possibility of being more focused and gathered) • Categorizing experiences *v.* describing bare sensations/thoughts • Getting to know the territory of schizophrenia	i. Seeing/hearing and intentional awareness of breath, body, sounds and thoughts ii. 3-min breathing space (awareness of body, re-directing and expanding attention), opening (controlling breath) iii. Stretching and breathing (homework) iv. Walking and focused sensation v. Yoga (homework)
		Session 3: Acceptance, holding, allowing; and letting be Rationale: a. Relating differently involves bringing to the experience a sense of allowing it to be as it is, without judging it or trying to make it different; and b. An accepting attitude is a major part of taking care of oneself and seeing clearly what, if anything, needs to change	• Allowing and accepting attitude towards the illness and its symptoms • Awareness of and opening up troubles in the mind; expanded breathing and stress-holding space	i. Exercise on awareness of breath, body, thoughts and emotions ii. Recognizing and discussing difficulties with such awareness iii. Expanded breathing space – opening up troubles in the mind and settling down these troubles iv. Mindful walking
	3. Empowerment of self-control of psychotic symptoms and negative thoughts (one session)	a. Negative thoughts and moods that accompany them colour or reduce our ability to relate to experiences; b. Thoughts are merely thoughts, we can choose whether to engage with them or not; and c. The same patterns of thoughts recur again and again, without necessarily having to question them and seek alternatives	• Thoughts are not facts – alternative perspectives of seeing your thoughts and sensations • Options for working with negative and disorganized thoughts • Recognizing the recurring thoughts and standing back from them, without questioning them	i. Expanded breathing space ii. Alternative perspectives and options for working with thoughts iii. Diary writing and awareness of early warning signs of relapse iv. Selection of practices (homework)
II.	1. Knowledge of schizophrenia and its care (two sessions)	a. Understanding psychotic symptoms and individual psychosocial health concerns; b. Understanding cultural issues within family and society; and c. Identifying important needs for patients, self and family	• Patients’ individual health needs in relation to schizophrenia care • Information sharing of schizophrenia and its treatment • Sharing of behavioural and perceptual problems, intense emotions, and feelings about illness management • Discussing ways to deal with negative thoughts and emotions, cultural issues and beliefs of mental illness, stigma and family • Information about medication and its effects, self-care, daily activities and functioning, and illness and home management	i. Group discussion and video watching ii. Information search from Internet and health care organizations iii. Expert (both ex-patients and professionals) sharing iv. Selection of mindfulness practices learned (homework) v. Communication and social skills training
	2. Illness management and problem solving (one session)	a. Information about self-management of schizophrenia and its related behavioural problems; and b. Learning effective coping and problem-solving skills	• Enhancing social support, stress coping and problem-solving skills by working on each member's life situations • Performing behavioural rehearsals of social interactions with co-patients (and invited family members) within groups • Review of real-life practice of coping skills learned in group sessions	i. Group discussion and video watching ii. Ex-patients’ sharing of illness management experiences iii. Role play on coping and problem-solving skills iv. Practices of coping skills learned (homework)
III.	1. Behavioural rehearsal of relapse prevention (two sessions)	Session 1: How can I best take care of myself? Rationale: a. Specific things can be done when psychotic symptoms threaten my living and functioning; b. Taking a breathing space first and then deciding what action to take; c. Each patient has his/her own unique patterns of symptoms and relapse and thus also his/her own prevention strategies; and d. Group members can provide support and help each other to plan the best self-care	• Identifying signs of relapse and associated factors • Reflect on daily activities, stressors and accompanying emotions (i.e. nourishing *v.* depleting activities) • Evaluation of self-care, illness management, coping skills and interpersonal relationships	i. Group discussion ii. Role play and behavioural rehearsals of coping skills and self-reflection iii. Awareness of breath, body, sounds, thoughts, difficulty, and social support iv. Breathing space and selecting forms of practice to continue v. Continuous practice of coping skills learned (homework)
		Session 2: Using learned skills to deal with future problems in thoughts and moods Rationale: a. Maintaining balance in life is helped by regular mindfulness practice; and b. Good intentions can be strengthened by linking practice with positive thoughts and reasons for taking care of oneself	• What thing(s) in our life do you value most and what can the practice help you with? • Preparing for future life problems and relapse prevention • Consolidation of selected and practiced coping and mindfulness skills	i. Body scan, sitting and walking mindfulness ii. Best wishing and positive thinking iii. Group discussion about future problems iv. Continuous practice of selected mindfulness strategies (homework)
	2. Community resources and future plans (one session)	a. Being familiar with community support services and resources for schizophrenia care; b. Review of main issues and those skills learned and selected for practices; and c. Planning for future independent living	• Summary of the main issues and topics covered and knowledge and skills learned • Introduction of available community support resources • Issues expected in future life and psychological and behavioural preparations for the future • Action plans for illness management and the future • Questions and comments from group members and specific requests for follow-up	i. Body scan and mindful walking ii. Discussion about learning from the programme and plan for the future iii. Checking each person's support resources/mechanisms iv. Invitation to outcome assessment and interviews

MBPEG, Mindfulness-based psychoeducation group programme.

The MBPEG adopted some additional specific strategies to address commonly held Chinese cultural attitudes. During the first phase, the group sessions focused on understanding their strong interdependence, encouraging better problem-solving and mutual support between group members. Within the second/third phases, participants were encouraged to cultivate an open and accepting attitude and positive responses to life events or problems and to develop a ‘decentred’ (i.e. passing events in mind) attitude on their thoughts or related feelings (Ma & Teasdale, 2004; Chien & Lee, 2013). During the later sessions, they were supported to explore the strong self-centredness that is traditionally common in some Chinese cultural groups (e.g. ‘saving face’ and self-blame) and accordingly reconstruct their self-image and perception.

Similar to the MBPEG, the CPEG (*n* = 114) consisted of 12 bi-weekly 2-h sessions (10–12 participants per group), which was based on previously developed group psychoeducation manuals (Macpherson *et al.* 1996; Chien & Bressington, 2015). Participants received psychosocial support and psychoeducation from one of three psychiatric nurses trained by the research team and one psychotherapist within a 3-day workshop. The trained psychiatric nurses were already well experienced in facilitating psychiatric rehabilitation and education groups (average years of working in psychiatric rehabilitation services: 7.5 years). CPEG comprised of four stages: joining with individual patients and families (two sessions, focusing on orientation and engagement of participants and discussion about their goals and action plans for illness management); an education and survival skills workshop (four sessions, mainly concerning basic information about schizophrenia and related behaviours, uncovering common stressful situations, and exploring effective coping strategies); problem-solving training to help prevent relapse (four sessions); and review on their learned knowledge and skills and preparing for the future (two sessions).

All group sessions of MBPEG and CPEG were audio-recorded and progress monitoring of the two programmes was made between group sessions by reviewing all audiotapes. Fidelity to treatment was assessed with a checklist as suggested by the NIH Behavior Change Consortium recommendations (Bellg *et al.* 2004). A researcher scored each recording using the checklist. The fidelity scores ranged from 90.2 to 95.2% with a mean of 92.8% for the MBPEG run by the six advanced practice nurses and from 90.0 to 94.0% for the CPEG run by the three psychiatric nurses. There were no significant differences found on the fidelity scores between the therapists (*p* values >0.10) and the six clinics under study (*p* = 0.18) using the Kruskal–Wallis test.

TAU consisted of routine psychiatric out-patient services received by the participants (*n* = 114), which were similar across the six clinics. These services primarily consisted of regular appointments (every 4 to 6 weeks) with psychiatrists. The TAU also involved contacts with nursing staff (monthly; after medical consultation) where nursing advice and brief education were given about the illness/treatments and community mental healthcare services. Whenever necessary, social welfare and finance assistance was offered by medical social workers and psychological treatment was provided by clinical psychologists. Other structured psychological interventions received during the study period were reported by the participants and counter-checked with the out-patient records of the clinics; however, there was not any MBI used in TAU.

### Outcomes

Outcome assessments were conducted at participant recruitment and at 1 week and 6, 12 and 24 months post-treatment by research nurses (one in each country, trained in the use of the measures) who were blind to the group/intervention assignment. The primary outcome measures were average number and length of re-hospitalizations over the previous 6 months and the 30-item PANSS (Kay *et al.* 1987). Complete remission was defined as 4-month simultaneous ratings of all individual items in PANSS as score ⩽3 (Andreasen *et al.* 2005).

Secondary outcomes included level of functioning as measured by the Specific Level of Functioning Scale (SLOF) (Schneider & Struening, 1983) and insight into illness/treatment (Insight and Treatment Attitudes Questionnaire; ITAQ) (McEvoy *et al.* 1989). These scales were validated Chinese versions (Chan *et al.* 2009; Chien & Thompson, 2014; Chien & Bressington, 2015). Patients’ demographic, clinical and treatment-related data were also collected at baseline. Dosages of antipsychotics were converted into haloperidol equivalents for comparison.

### Statistical analyses

Sample size was calculated based on the results of earlier trials of psychoeducation for Chinese patients with schizophrenia, where the primary outcomes were symptom severity and re-hospitalization rate (Chan *et al.* 2009; Chien & Lee, 2013). Study power calculation showed that 342 subjects (*n* = 114 per group) were required to identify any statistically significant differences on re-hospitalization rate and mental state between three groups (particularly between two psychoeducation groups) at an average effect size of 0.25, *p* = 0.05 and power of 0.80, and accounting for a potential attrition rate of 25%.

Based on the intention-to-treat principle, all analyses were carried out using IBM's SPSS version 20.0 (USA). Adequacy of randomization was assessed by between-group and between-country comparisons of baseline sociodemographic data and outcome measures (PANSS, SLOF, ITAQ, and re-hospitalization and symptom remission rates), using χ^2^ tests for dichotomous variables, and Kruskal–Wallis statistics and analysis of variance (ANOVA) tests for ordinal and interval data, respectively. Ensuring no violations of assumptions of normality, linearity and homogeneity of variance–covariance, and multi-collinearity, a mixed-model multivariate ANOVA (MANOVA) test was performed for the dependent (outcome) variables to determine whether the interventions produced the within-between group and interactive group × time effects hypothesized, followed by univariate analyses (repeated-measures ANOVA tests) of the variables if the MANOVA test results were found to be significant. Helmert contrasts codes were set to test any significant between-group differences on those measures with significant results in the ANOVA tests. Within-group differences of the MBPEG participants between six clinics, three countries and low (<6 sessions) and high (⩾6 sessions) attendees were examined on those outcomes with significant results in MANOVA and repeated-measures ANOVA tests.

Complete remissions as the categorical outcome measures were presented as conditional odd ratios (ORs) and were best fitted by a logistic proportional odds random intercepts and slopes model. Mixed-effects multilevel models were used to analyse the overall significance of the model (Wald χ^2^ statistic) and modelled (intention-to-treat) group differences on remission rates at 24-month follow-up (i.e. whether MBPEG and/or CPEG plus TAU was better or worse than TAU alone at the last follow-up time-point). With only a few missing data over the 24-month follow-ups, the last data were brought forward for data imputation. The level of statistical significance was set at 0.05, except the univariate ANOVA tests at 0.01 (i.e. Bonferroni's correction of *α* level).

### Ethical standards

All procedures contributing to this work comply with the ethical standards of the relevant national and institutional committees on human experimentation and with the Helsinki Declaration of 1975, as revised in 2008.

## Results

Recruitment took place between 1 October 2013 and 30 April 2014 and all follow-up assessments were completed by a research assistant who was blind to subject recruitment and intervention assignment. Of the 300 (30%) patients screened for eligibility in each country, 240 (80% in Hong Kong), 210 (70% in China) and 208 (69% in Taiwan) were found eligible and agreed to participate. About 30–35 patients in each country (12–17%) were approached but refused to participate, mainly due to lack of interest in the study or programme participation (*n* = 13–15), reluctance to talk about the illness (*n* = 10–12) and/or having concerns about time inconvenience for attending the intervention (*n* = 7–11). There were 35, 36 and 40 patients in the three countries who, after screening, withdrew from participating in this study and their demographic and clinical characteristics did not show significant difference from those of the participants in this study. Table 2 summarizes the demographic and clinical characteristics of the 342 patients in the three study groups (*n* = 114 in each group) and the non-participants (*n* = 388) who were eligible but not selected in the study. There were no significant differences in sociodemographic characteristics or antipsychotic medications between the study groups and the non-participants at baseline.

**Table 2.** Baseline sociodemographic and clinical characteristics of the MBPEG, conventional psychoeducation and usual-care groups and the non-participants

Characteristics	MBPEG (*n* = 114)	CPEG (*n* = 114)	TAU (*n* = 114)	Non-participants (*n* = 316)	Test value^a^	*p*
Gender, *n* (%)					1.50	0.22
Male	72 (63.2)	70 (61.4)	74 (64.9)	190 (60.1)		
Female	42 (36.8)	44 (38.6)	40 (35.1)	126 (39.9)		
Mean age, years (s.d.)	25.1 (6.8)	25.8 (7.9)	26.0 (8.5)	25.9 (12.8)	1.92	0.11
18–29, *n* (%)	38 (33.3)	37 (32.5)	36 (31.6)	112 (35.4)		
30–39, *n* (%)	44 (38.6)	42 (36.8)	48 (42.1)	110 (34.8)		
40–49, *n* (%)	32 (28.1)	35 (30.7)	30 (26.3)	94 (29.8)		
Education level, *n* (%)					1.89	0.14
Primary school or below	26 (22.8)	28 (24.6)	23 (20.2)	80 (25.3)		
Secondary school	68 (59.7)	65 (57.0)	70 (61.4)	185 (58.6)		
University or above	20 (17.5)	21 (18.4)	21 (18.4)	51 (16.1)		
Primary diagnosis, *n* (%)					1.21	0.20
Schizophrenia	61 (53.5)	58 (50.9)	59 (51.8)	188 (59.5)		
Schizophreniform disorder	15 (13.2)	14 (12.3)	12 (10.5)	33 (10.4)		
Schizo-affective disorder	25 (21.9)	26 (22.8)	27 (23.7)	61 (19.3)		
Other psychotic disorders	13 (11.4)	16 (14.0)	16 (14.0)	34 (10.8)		
Mean monthly household income, HKD^b^ (s.d.)	15 130 (3781)	14 075 (4105)	14 887 (4870)	17 012 (5976)	1.90	0.12
5000–10 000, *n* (%)	15 (13.2)	12 (10.5)	14 (12.3)	45 (14.2)		
10 001–15 000, *n* (%)	38 (33.3)	39 (34.2)	37 (32.5)	115 (36.4)		
15 001–25 000, *n* (%)	36 (31.6)	37 (32.5)	38 (33.3)	110 (34.8)		
25 001–35 000, *n* (%)	25 (21.9)	26 (22.8)	25 (21.9)	46 (14.6)		
Mean duration of illness, years (s.d., range)	2.6 (2.1, 0.25–5.0)	2.5 (1.7, 0.5–4.5)	2.7 (1.9, 0.5–5.0)	2.6 (2.4, 0.25–5.0)	1.95	0.15
<1, *n* (%)	25 (21.9)	21 (18.4)	20 (17.5)	71 (22.5)	1.89	0.12
1–2, *n* (%)	33 (28.9)	35 (30.7)	32 (28.1)	117 (37.0)		
2–3, *n* (%)	35 (30.7)	34 (29.8)	36 (31.6)	95 (30.1)		
3–5, *n* (%)	21 (18.4)	24 (21.1)	26 (22.8)	63 (19.9)		
Number of family members living with patient, *n* (%)						
0–1	28 (24.6)	25 (21.9)	24 (21.1)	95 (30.1)	2.01	0.10
2–3	58 (50.8)	55 (48.2)	59 (51.7)	175 (55.4)		
4–5	28 (24.6)	34 (29.8)	31 (27.2)	46 (14.6)		
Use of psychiatric services, *n* (%)						
Medical consultation and treatment planning	113 (99.1)	114 (100.0)	113 (100.0)	313 (99.0)	1.40	0.22
Nursing advice on services and brief education	70 (61.4)	62 (54.4)	68 (59.7)	201 (63.6)		
Social welfare and financial advices	70 (61.4)	66 (57.9)	69 (60.5)	210 (66.5)		
Individual/family counselling	30 (26.3)	29 (25.4)	30 (26.3)	79 (25.0)		
Type of medication, *n* (%)						
Conventional antipsychotics (e.g. haloperidol)	30 (26.3)	31 (27.2)	29 (25.4)	90 (28.5)	1.44	0.21
Atypical antipsychotics (e.g. risperidone)	40 (35.1)	38 (33.3)	39 (34.2)	130 (41.1)		
Anti-depressants (e.g. fluoxetine)	14 (12.3)	11 (9.7)	10 (8.8)	30 (9.5)		
Blended mode^c^	30 (26.3)	34 (29.8)	36 (31.6)	66 (20.9)		
Dosage of medication^d^, *n* (%)						
High	24 (21.1)	21 (18.4)	22 (19.3)	72 (22.8)	1.98	0.13
Medium	65 (57.0)	68 (59.7)	69 (60.5)	184 (58.2)		
Low	25 (21.9)	25 (21.9)	23 (20.2)	60 (19.0)		

MBPEG, Mindfulness-based psychoeducation group; CPEG, conventional psychoeducation group; TAU, treatment as usual; s.d., standard deviation; HKD, Hong Kong dollar; df, degrees of freedom.

a An analysis of variance (*F* test, df = 656) or the Kruskal–Wallis test by ranks (*H* statistic, df = 3) was used to compare the sociodemographic variables of patients among the three study groups and the non-participants.

b US$1 = HK$7.80.

c Patients were taking more than one type of psychotropic medication such as the use of either conventional and atypical antipsychotic or atypical antipsychotic together with one anti-depressant.

d Dosage levels of neuroleptic/antipsychotic medications were compared with the average dosage of medication taken by the patients in haloperidol-equivalent mean values.

The study procedure and patient flow are presented in Fig. 1. Attritions over the 6-month treatment and 2-year follow-up periods were relatively low at 16–17% in the three study groups. Two CPEG participants during the follow-up period and one in the TAU group during the treatment period died due to chronic medical diseases, which were found unrelated to the interventions used. There were also no reported or observed adverse effects of the interventions. There were 10 and 13 participants who attended <6 (<50%) group sessions of the MBPEG and CPEG, respectively. Only limited amounts of data were missing across all measurements, with 2% of primary and 5% of secondary outcomes not being available. There was no difference in the distribution of item/measure completion between groups (χ^2^ = 1.50, degrees of freedom = 2, *p* = 0.23). The three groups, and three countries under study, did not differ significantly on any baseline mean scores of the outcome variables and indicating minimal covariate effects.

Results of Box's and Levene's tests indicated no violations of the assumptions of homogeneity of variance–covariance matrices (*p* = 0.18) and equality of variances of the outcome variables between groups (*p* = 0.25), respectively. There were also few outliers, satisfactory multivariate normality and moderate correlations between the outcome measures (*r* = 0.20–0.46), supporting the use of the MANOVA test to compare the outcome scores between groups across the five assessment points (times 1–5). Results of mixed-model MANOVA indicated a significant interactive (group x time) treatment effect or a statistically significant difference between the three study groups (*F*_5,340_ = 8.95, *p* = 0.0005; Wilks’ *λ* = 0.98, partial *η*^2^ = 0.34). When the results (between-group effects) for each of the outcome variables were considered separately (as shown in Table 3), the three groups indicated a significant difference on reduction in PANSS score (*p* = 0.003) and average length/duration of re-hospitalizations (*p* = 0.005), and improvement in level of functioning (SLOF score, *p* = 0.002) and insight into the illness/treatment (ITAQ score, *p* = 0.0008), using a Bonferroni's adjusted α of 0.01. An analysis of the adjusted mean scores at times 1–5 indicated that the MBPEG participants reported better progressive improvements in their length of re-hospitalizations (*p* = 0.05), symptom severity (*p* = 0.01), insight into illness/treatment (*p* = 0.005) and functioning (*p* = 0.01) than those in the CPEG. The TAU group reported progressive mild to moderate deteriorations in most of the outcome scores over the 24-month follow-up. Statistically significant differences were also found on the three SLOF subscales scores (self-maintenance, social functioning and community living skills; *p* = 0.002–0.005), and PANSS (positive and negative symptoms; *p* = 0.003 and 0.01, respectively) between the three groups across the follow-up period.

**Table 3.** Outcome measure scores at pre-test and four post-tests, results of MANOVA (group × time) and remission rates (*n* = 265)

	MBPEG (*n* = 111)					CPEG (*n* = 110)					TAU (*n* = 112)					
Instrument	Time 1	Time 2	Time 3	Time 4	Time 5	Time 1	Time 2	Time 3	Time 4	Time 5	Time 1	Time 2	Time 3	Time 4	Time 5	*F*_2,330_
ITAQ (0–22)^a^	9.3 (3.1)	10.5(4.7)	13.9 (5.1)	14.3 (6.3)	17.9 (5.9)	9.5 (3.3)	9.0 (4.9)	10.5 (5.8)	9.8 (6.1)	10.1 (6.3)	9.5 (4.3)	9.0 (5.3)	9.8 (5.9)	9.0 (4.9)	8.9 (6.8)	8.98***
SLOF (43–215)^a^	139.1 (17.8)	158.0 (18.1)	169.2 (19.0)	177.9 (20.1)	198.1 (25.0)	137.9 (18.8)	143.8 (21.1)	146.5 (20.4)	149.9 (21.4)	156.1 (19.1)	138.8 (16.1)	136.4 (17.1)	134.9 (17.3)	130.4 (17.8)	132.1 (20.1)	6.40**
Self maintenance	42.6 (8.1)	45.8 (8.1)	49.8 (9.5)	52.3 (10.9)	57.8 (11.0)	41.8 (9.3)	43.1 (9.1)	44.7 (9.0)	44.4 (9.2)	46.2 (9.8)	41.4 (8.2)	39.4 (8.9)	37.9 (9.0)	35.4 (7.2)	36.0 (8.0)	5.83**
Social functioning	42.4 (8.5)	46.8 (9.0)	52.9 (9.7)	54.3 (11.0)	59.5 (11.5)	45.5 (9.0)	45.8 (9.0)	47.2 (8.7)	48.6 (9.2)	49.1 (10.2)	42.9 (9.1)	41.9 (9.1)	41.2 (9.0)	39.5 (8.4)	39.3 (9.1)	6.79**
Community living skills	54.1 (9.8)	65.4 (10.1)	67.9 (10.3)	71.3 (11.2)	79.8 (12.0)	51.7 (10.5)	56.9 (9.3)	54.6 (7.7)	57.8 (10.2)	60.2 (12.4)	54.0 (8.9)	55.1 (8.5)	54.8 (11.0)	55.5 (9.6)	56.9 (9.0)	7.00**
PANSS (30–210)^a^	80.3 (10.5)	74.8 (8.9)	71.1 (10.0)	69.9 (9.2)	64.3 (8.0)	81.0 (8.7)	82.1 (9.2)	80.7 (8.5)	79.0. (9.9)	76.5 (9.1)	81.0 (9.2)	80.9 (8.7)	85.8 (9.8)	89.0 (12.3)	89.8 (14.0)	6.07**
Positive symptoms	20.1 (6.8)	17.1 (6.5)	15.2 (7.0)	13.0 (5.0)	12.0 (4.8)	20.5 (8.0)	20.3 (7.8)	19.0 (8.1)	18.5 (6.3)	17.9 (5.9)	20.1 (8.0)	19.8 (6.7)	20.9 (8.1)	21.8 (9.1)	23.8 (10.0)	6.48**
Negative symptoms	19.9 (7.3)	17.0 (7.0)	18.0 (6.2)	18.5 (5.2)	19.0 (6.0)	19.5 (8.3)	19.9 (8.9)	18.9 (9.2)	19.0 (10.5)	19.1 (11.8)	20.1 (9.1)	20.8 (9.6)	21.8 (9.8)	21.3 (10.7)	22.5 (11.7)	5.10*
Re-hospitalizations																
Average number^b^	2.9 (1.8)	2.5 (1.5)	2.3 (2.0)	2.2 (1.5)	1.9 (1.6)	3.0 (2.0)	2.9 (1.4)	2.7 (2.0)	2.7 (1.8)	2.6 (2.0)	3.0 (1.6)	2.7 (1.6)	2.8 (2.4)	3.0 (2.2)	2.8 (2.3)	3.78
Duration^c^, days	19.2 (8.3)	15.6 (5.1)	13.2 (6.6)	12.0 (7.0)	10.1 (7.1)	18.9 (8.0)	17.7 (9.1)	16.2 (9.1)	17.5 (9.0)	16.1 (10.0)	19.2 (9.1)	21.8 (9.8)	20.2 (10.2)	21.0 (9.9)	21.9 (12.9)	5.23**
Remission rate^d^, %	–	9.5	15.1	29.7	38.9	–	8.9	13.0	24.8	27.0	–	6.9	8.7	9.8	7.4	–
χ^2^ (*p*)^e^	–	1.02 (0.50)	3.0 (0.09)	4.9 (0.01)	5.9 (0.001)	–	0.94 (0.55)	2.8 (0.09)	4.3 (0.03)	4.0 (0.01)	–	–	–	–	–	–
RR^f^ (95% CI)	–	1.3 (0.5–2.4)	1.7 (0.7–3.0)	2.5 (1.3–4.9)	2.0 (1.1–4.2)	–	1.2 (0.4–0.2)	1.6 (0.6–2.8)	2.9 (1.1–4.1)	2.8 (0.7–3.1)	–	–	–	–	–	–
NNT	–	35	9.6	5.4	3.4	–	36	10.3	6.5	5.0	–	–	–	–	–	–

Data are given as mean (standard deviation) unless otherwise indicated.

MANOVA, Multivariate analysis of variance; MBPEG, mindfulness-based psychoeducation group; CPEG, conventional psychoeducation group; TAU, treatment-as-usual; time 1, baseline measurement at the start of intervention; time 2, 1-week post-intervention; time 3, 6 months post-intervention; time 4, 12 months post-intervention; time 5, 24 months post-intervention; ITAQ, Insight and Treatment Attitudes Questionnaire; SLOF, Specific Level of Functioning Scale; PANSS, Positive and Negative Syndrome Scale; RR, relative risk; CI, confidence interval; NNT, number needed to treat.

a Possible range of scores of each scale indicated in parentheses.

b Average number of readmissions to a psychiatric in-patient unit over the previous 6–12 months at the five measurements (times 1 to 5).

c Duration/length of readmissions to a psychiatric in-patient ward/unit in terms of average number of days of hospital stay over the previous 6 months at times 1 to 5.

d Complete remission rates defined as 4-month simultaneous ratings of all PANSS item scores ⩽3.

e χ^2^ Frequency between MBPEG and TAU, CPEG and TAU.

f RR using TAU as reference.

* *p* < 0.01, ** *p* < 0.005, *** *p* < 0.001.

The Helmert's contrasts tests indicated that the mean differences of the following outcomes between the MBPEG and the psychoeducation or routine-care group between two time points were significant at 0.05 and contributed to the overall multivariate significance:

(a) Average length of re-hospitalizations in the MBPEG was significantly greater reduced from times 1 to 4, whereas it was slightly reduced in the CPEG and consistently increased in routine care over time;
(b) Patients’ functioning in the MBPEG was significantly greater improved from times 1 to 4; and for the CPEG, it also consistently improved over time and differed significantly from that of the patients in routine care who showed a consistent deterioration of functioning over time;
(c) Patients’ mental state in the MBPEG was also significantly greater improved from times 1 to 4; and for the CPEG, it was improved and differed significantly from that in routine care at times 2 to 3; and
(d) Insight into illness score of the MBPEG was significantly greater enhanced from times 1 to 4 than the other two groups, whereas for the CPEG it increased slightly over time.

The above significant outcomes (ITAQ, SLOF, PANSS and length of re-hospitalizations) did not yield significant differences between the MBPEG participants in the six clinics (*F*_1,110_ = 2.13–2.81, *p* = 0.15–0.28) and in groups of patients with low (<6 sessions) and high (⩾6 sessions) attendance of the intervention (*F*_1,105_ = 2.15–3.45, *p* = 0.10–0.19). Appendix table in the additional information summarizes the mean scores and standard deviations of the outcome measures of the participants in the three study groups at the six clinics over the follow-up.

Complete remissions (4-month simultaneous ratings of all PANSS item scores ⩽3) were not significantly more likely in the MBPEG and CPEG than in the TAU group at times 2 and 3, but significant differences emerged during times 4 and 5. In addition, there was a significant difference on remission rate at the latest post-test between the MBPEG and CPEG [at 24 months: 38.9% *v.* 27.0%, χ^2^ = 4.1, *p* = 0.010, relative risk (RR) = 1.9, 95% confidence interval (CI) 0.9–3.0, number needed to treat (NNT) = 2.0]. However, there were no significant differences on these rates between the three study groups across the six clinics or three countries (*p* = 0.12–0.38).

The differences on the estimated odds between the two intervention groups, and between the MBPEG and TAU-alone group, were significant at 12 months (Δ = 1.6 and 1.3, 95% CI 0.58–2.83 and 0.45–2.31, *p* = 0.022 and 0.039) and 24 months follow-up (Δ = 2.9 and 1.9, 95% CI 0.98–4.38 and 0.65–2.89, *p* = 0.001 and 0.010). Mixed-effects logistic regression indicated a significant differential change in proportional ORs over the 24-month follow-up; 37 and 26% of the MBPEG and CPEG participants, respectively, but only 7.2% of the TAU group were in remission (χ^2^ = 8.9 and 8.0, *p* = 0.001 and 0.003, RR = 3.5 and 3.1, 95% CI 2.0–7.2 and 1.6–6.3, NNT = 3.2 and 4.6).

There were no significant differences in the medication dosages based on the converted haloperidol equivalents between MBPEG and CPEG groups (*F*_1,220_ = 3.70, *p* = 0.10). Similarly, no significant differences were found across the three groups in psychotropic medications used and types, frequency or hours of participation in other individual- or family-based psychosocial interventions or psychiatric treatments (Kruskal–Wallis statistics, *p* = 0.12–0.25).

## Discussion

This is the first international multi-centre RCT of an MBI for people with schizophrenia. Improvements in re-hospitalization, psychotic symptoms, functioning and insight into the illness/treatment were comparable between the MBPEG and CPEG at post-treatment or short-term (6 months) follow-up, while significant differences between the MBPEG and the other two study groups (the CPEG and TAU-alone group) were found on these outcomes over 12- and 24-month follow-ups in all three countries. Indeed, the improvements in patient outcomes in the MBPEG were enhanced and sustained over time, whilst the TAU-alone group deteriorated progressively in most patient outcomes during the follow-up. It appears that the MBPEG with combined mindfulness training and components of psychoeducation has indicated more sustainable and greater benefits to these patients than the conventional psychoeducation programme alone.

The findings also indicated that there was progressive enhancement of remission rates in the participants receiving MBPEG and those receiving CPEG over the 24-month follow-up. Nevertheless, significant greater symptom reduction and thus complete remission rates were observed among the participants receiving MBPEG when compared with the other two study groups at both 12- and 24-month follow-up. At 24-month follow-up, more than one-third of the participants receiving MBPEG were in complete remission, compared with about 26 and 7% of those receiving CPEG and TAU alone, respectively.

The effect sizes observed in this study are generally moderately large, particularly on length of re-hospitalizations, positive symptoms, psychosocial functioning and insight into the illness/treatment. The short-term patient outcomes of the MBPEG demonstrated larger effect sizes when compared with those reported in recent literature reviews (Khoury *et al.* 2013), and those recommended by the US treatment guidelines (Lehman *et al.* 2004). Previous studies that indicated more significant effects (e.g. Chadwick *et al.* 2009) have included small-sized samples with a longer duration of the illness, been conducted <12 months in a single study site, and used different combined approaches to cognitive therapy. Further multi-site controlled trials, involving comparative treatments such as cognitive–behavioural therapy, cognitive re-mediation and/or other insight-inducing therapies, and/or shorter *v.* longer duration of treatment, are recommended to establish the specificity and superiority of the intervention and its positive effects reported in this study.

As hypothesized, the differences on most patient outcomes between the MBPEG and TAU-alone group increased during the 12-month follow-up. A controlled study of a mindfulness education group programme has reported similar effects of symptom reduction and increased mindful responses to stressful events in patients with less chronic psychotic disorders immediately after intervention (Langer *et al.* 2012). Another single-site controlled trial of a similar mindfulness-based psychoeducation programme in Hong Kong has also showed a similar pattern of therapeutic gains with Chinese people with schizophrenia at both 12- and 24-month follow-up (Chien & Thompson, 2014).

No adverse effects related to the MBI were found in this study. Therefore, concerns previously expressed in the literature that mindfulness and meditation training for people with psychosis may exacerbate psychotic symptoms (Chadwick *et al.* 2009; Chien & Lee, 2013) were not supported by this study. It is also important and interesting to note that the substantive significant improvements of patients could be contributed by a combined effect of mindfulness training and psychoeducation, and their core elements (e.g. self-empowerment and regulation for illness management). Many of these core elements can be considered potential therapeutic components in current models of psychosocial interventions (Chien *et al.* 2006; Chan *et al.* 2009). Therefore, further research is recommended to explore the possible benefits of individual components of the MBPEG.

While this controlled trial has strong internal validity using a random sample from three countries, high treatment fidelity and moderately long follow-up, a few main limitations should be noted. First, the participants were motivated to participate and were not blind to the intervention allocation, which might produce an expectation or response bias. Second, patients with a shorter duration of schizophrenia (mean = 2.5–2.7 years), as in this study, might not be representative of the wider schizophrenia population, those with chronic schizophrenia, or those with co-morbidities of other mental disorders such as substance misuse and affective disorders (Chien & Thompson, 2014; Chien & Bressington, 2015). Therefore, this selective sample might have contributed to high levels of adherence to the intervention, good attendance to intervention sessions and the very low attrition rate in this study. Third, this study was conducted by advanced practice nurses who received intensive training from the researchers, which may reduce its applicability into usual psychiatric care that tends to favour brief and easy-to-run therapies with simple training. Lastly, these positive patient outcomes were generated by psychiatric out-patient services in culture-specific Chinese contexts. As mindfulness principles are traditionally commonplace in many Asian countries it is possible that the MBPEG intervention may be more acceptable to patients in Chinese contexts, which may limit the generalizability of our findings. Further multi-centre controlled trials with psychotic patients from diverse ethnic and sociodemographic characteristics are therefore recommended. Considering that schizophrenia/psychosis is a long-term condition, future research should also consider the longer-term benefits (e.g. over 2 years) of approaches to MBI in people with psychosis.

Notwithstanding its limitations, this was the first multi-centre trial to test a mindfulness-based psychoeducation programme for people with schizophrenia over a 24-month follow-up. Compared with a conventional psychoeducation group programme and usual care alone, the patients receiving the MBPEG indicated significant reduction of psychotic symptoms and duration of re-hospitalizations, and improvements in functioning and insight into the illness/treatment over 24 months post-intervention.

## Appendix

**Table:** Outcome measure scores at pre-test and four post-tests for three study groups in three countries (*N* = 265)

	MBPEG (*n* = 111)			CPEG (*n* = 110)			TAU (*n* = 112)		
	Hong Kong	China	Taiwan	Hong Kong	China	Taiwan	Hong Kong	China	Taiwan
Instrument	M ± s.d.	M ± s.d.	M ± s.d.	M ± s.d.	M ± s.d.	M ± s.d.	M ± s.d.	M ± s.d.	M ± s.d.
ITAQ (0–22)^a^									
Time 1	9.3 ± 3.0	9.4 ± 3.1	9.2 ± 3.0	9.3 ± 3.5	9.1 ± 3.4	9.5 ± 3.7	9.3 ± 4.0	9.2 ± 3.9	9.2 ± 4.0
Time 2	10.3 ± 4.4	10.5 ± 4.2	10.2 ± 4.9	9.2 ± 4.6	9.5 ± 3.9	9.1 ± 3.5	9.1 ± 5.0	9.0 ± 4.0	9.2 ± 4.1
Time 3	13.7 ± 5.0	13.6 ± 5.4	13.8 ± 5.9	10.3 ± 5.3	10.2 ± 5.0	10.6 ± 5.8	9.7 ± 5.1	9.8 ± 5.0	9.7 ± 4.3
Time 4	14.1 ± 6.0	14.4 ± 6.9	14.0 ± 6.5	9.9 ± 6.0	9.8 ± 5.1	10.0 ± 5.9	9.1 ± 4.0	8.9 ± 3.7	9.2 ± 3.9
Time 5	17.7 ± 5.3	17.9 ± 5.8	17.5 ± 6.5	10.2 ± 6.0	10.4 ± 6.1	10.2 ± 6.7	8.8 ± 2.9	9.0 ± 3.0	9.1 ± 4.0
SLOF (43–215)									
Time 1	139.0 ± 17.1	139.1 ± 15.8	138.9 ± 17.0	137.5 ± 18.2	137.8 ± 17.4	138.2 ± 18.1	138.7 ± 16.2	138.6 ± 16.5	138.9 ± 16.2
Time 2	158.8 ± 17.0	159.1 ± 15.8	159.1 ± 14.9	143.6 ± 16.9	143.9 ± 17.8	143.8 ± 19.0	136.5 ± 16.4	136.7 ± 17.2	136.3 ± 18.1
Time 3	168.8 ± 19.8	169.1 ± 16.8	169.8 ± 18.9	146.7 ± 19.6	146.1 ± 20.0	146.8 ± 20.2	134.7 ± 17.9	134.6 ± 18.0	135.0 ± 18.2
Time 4	178.1 ± 19.8	179.1 ± 19.0	179.5 ± 17.0	149.5 ± 20.9	149.7 ± 21.0	150.3 ± 22.0	130.1 ± 18.4	130.7 ± 18.0	131.0 ± 19.2
Time 5	198.8 ± 19.8	198.0 ± 20.1	198.3 ± 20.3	156.2 ± 18.9	156.5 ± 21.2	156.0 ± 22.0	132.0 ± 18.1	131.8 ± 19.8	132.4 ± 19.1
PANSS (30–210)									
Time 1	80.2 ± 10.2	80.1 ± 10.0	80.5 ± 11.2	80.8 ± 8.6	81.2 ± 8.1	81.4 ± 8.8	80.9 ± 9.1	81.3 ± 9.0	81.0 ± 8.9
Time 2	74.6 ± 9.1	74.7 ± 8.8	74.9 ± 9.3	82.0 ± 8.9	82.4 ± 9.0	82.3 ± 9.2	80.8 ± 8.9	81.2 ± 9.0	81.0 ± 8.1
Time 3	71.0 ± 9.9	70.9 ± 10.1	71.3 ± 11.2	80.6 ± 8.1	80.9 ± 9.0	81.2 ± 9.2	85.6 ± 10.0	85.9 ± 10.2	85.4 ± 8.8
Time 4	69.7 ± 9.6	70.0 ± 9.7	70.3 ± 9.8	79.2 ± 10.0	78.6 ± 9.8	79.3 ± 9.5	89.3 ± 12.0	89.1 ± 11.8	88.7 ± 10.7
Time 5	64.2 ± 8.2	64.5 ± 8.8	64.6 ± 9.0	76.4 ± 9.0	76.3 ± 9.5	76.8 ± 9.8	89.7 ± 13.9	89.9 ± 13.1	90.0 ± 14.1
SSQ6 (0–30)									
Time 1	6.7 ± 2.0	6.7 ± 2.1	6.9 ± 2.2	7.0 ± 2.2	7.0 ± 2.3	7.4 ± 2.4	6.9 ± 2.6	7.1 ± 2.8	7.2 ± 2.9
Time 2	7.0 ± 2.0	6.7 ± 2.0	6.8 ± 2.1	7.0 ± 2.3	7.1 ± 2.9	7.4 ± 3.0	6.8 ± 2.9	7.0 ± 3.0	6.8 ± 3.2
Time 3	6.4 ± 2.5	6.6 ± 2.1	6.7 ± 2.5	6.7 ± 2.5	6.9 ± 2.3	7.0 ± 2.9	7.1 ± 3.4	7.2 ± 3.0	6.9 ± 3.0
Time 4	6.8 ± 2.4	6.7 ± 2.3	6.9 ± 2.8	7.0 ± 2.6	7.0 ± 2.5	7.1 ± 2.8	6.5 ± 3.0	6.9 ± 3.1	6.8 ± 3.0
Time 5	7.2 ± 2.4	7.1 ± 2.2	7.4 ± 2.3	7.1 ± 2.9	6.8 ± 3.0	6.8 ± 3.0	6.4 ± 2.2	6.7 ± 2.0	6.8 ± 2.9
Re-hospitalizations									
*Average Number*^b^									
Time 1	2.8 ± 1.9	2.9 ± 2.0	2.8 ± 3.0	3.1 ± 2.1	2.9 ± 2.1	2.9 ± 2.0	3.1 ± 1.8	3.0 ± 1.9	2.9 ± 2.0
Time 2	2.4 ± 1.9	2.6 ± 2.0	2.5 ± 2.4	2.9 ± 1.8	3.0 ± 2.2	2.8 ± 2.0	2.8 ± 1.5	2.6 ± 1.9	2.8 ± 1.9
Time 3	2.2 ± 2.1	2.4 ± 2.1	2.5 ± 2.1	2.8 ± 2.1	2.6 ± 3.0	2.7 ± 2.5	2.7 ± 2.0	2.8 ± 2.0	2.6 ± 2.0
Time 4	2.1 ± 1.9	2.3 ± 2.0	2.3 ± 2.0	2.8 ± 2.0	2.7 ± 2.1	2.9 ± 2.3	3.1 ± 2.5	2.9 ± 2.8	2.8 ± 2.2
Time 5	1.8 ± 1.5	2.0 ± 1.6	2.0 ± 1.7	2.6 ± 1.9	2.8 ± 2.0	2.7 ± 2.3	2.9 ± 2.2	2.7 ± 2.7	2.6 ± 2.0
*Duration*^c^									
Time 1	19.1 ± 8.0	19.3 ± 9.0	19.0 ± 9.1	18.8 ± 8.1	19.0 ± 8.8	19.0 ± 8.1	19.1 ± 9.0	19.4 ± 8.8	19.5 ± 9.2
Time 2	15.4 ± 5.5	15.8 ± 6.0	16.0 ± 8.2	17.6 ± 9.0	17.8 ± 9.0	17.8 ± 9.0	21.7 ± 10.0	21.7 ± 9.8	21.0 ± 10.0
Time 3	12.9 ± 6.9	13.5 ± 7.0	13.5 ± 7.1	16.3 ± 9.0	16.4 ± 10.0	16.1 ± 9.8	20.3 ± 10.0	20.4 ± 10.1	20.4 ± 9.0
Time 4	11.9 ± 6.9	12.2 ± 7.8	12.0 ± 7.0	17.3 ± 9.1	17.3 ± 9.0	17.7 ± 9.2	20.8 ± 9.8	20.9 ± 10.0	21.2 ± 10.5
Time 5	10.0 ± 7.1	10.3 ± 7.0	10.4 ± 7.8	16.2 ± 9.1	16.3 ± 9.3	16.0 ± 9.1	21.5 ± 13.0	21.9 ± 10.2	21.5 ± 10.2

MBPEG, Mindfulness-based psycho-education group; CPEG, conventional psycho-education group; TAU, treatment-as-usual; time 1, baseline measurement at the start of intervention; time 2, one-week post-intervention; time 3, 6 months post-intervention; time 4, 12 months post-intervention; time 5, 24 months post-intervention; ITAQ, Insight and Treatment Attitudes Questionnaire; PANSS, Positive and Negative Syndrome Scale; SSQ6, Six-item Social Support Questionnaire; SLOF, Specific Level of Functioning Scale.

a Possible range of scores of each scale indicated in parenthesis.

b Average number of readmissions to a psychiatric in-patient unit over the previous 6–12 months at the five measurements (times 1 to 5).

c Duration/length of readmissions to a psychiatric in-patient ward/unit in terms of average number of days of hospital stay over the previous 6 months at times 1 to 5.
