# An international multi-site, randomized controlled trial of a mindfulness-based psycho-education group program for people with schizophrenia – CORRIGENDUM

**DOI:** 10.1017/S0033291723000041

**Published:** 2023-04

**Authors:** W. T. Chien, D. Bressington, A. Yip, T. Karatzias

**Affiliations:** 1 Mental Health Care Research Group, School of Nursing, Faculty of Health and Social Sciences, The Hong Kong Polytechnic University, Hung Hom, Hong Kong, People's Republic of China; 2 School of Health & Social Care, Edinburgh Napier University, Edinburgh, UK

The authors clarify that the RCT reported in this Psychological Medicine article was a multi-centre RCT shared part of the data/results of another publication “**A randomized controlled trial of a mindfulness-based intervention program for people with schizophrenia: 6-month follow-up”** (Wang et al., 2016). Wang et al.'s (2016) report of the RCT is the results of one single study site (Hong Kong) of the same RCT as the international multi-site trial conducted in Hong Kong, mainland China and Taiwan (Registered at ClinicalTrials.gov; Ref: NCT01667601).
